# Preschool Confusion Assessment Method for the Intensive Care Unit–Spanish (psCAM-ICU-S): Cross-Cultural Adaptation and Validation in Colombia

**DOI:** 10.3389/fped.2021.749522

**Published:** 2021-12-07

**Authors:** Edwar Pinzón-Casas, Maira Soto-Trujillo, Laura Camargo-Agón, Ángela Henao-Castaño, Nathalie Gualdrón, Carolina Bonilla-González

**Affiliations:** ^1^Nursing Department, Pediatric Intensive Care Unit, Fundación Santa Fe de Bogotá University Hospital, Bogotá, Colombia; ^2^Department of Mental Health, Fundación Santa Fe de Bogotá University Hospital, Bogotá, Colombia; ^3^Subdirección de Estudios Clínicos y Epidemiología Clínica (SECEC), Fundación Santa Fe de Bogotá University Hospital, Bogotá, Colombia; ^4^Faculty of Nursing, Universidad Nacional de Colombia, Bogotá, Colombia; ^5^Department of Pediatrics, Pediatric Intensive Care Unit, Fundación Santa Fe de Bogotá University Hospital, Bogotá, Colombia

**Keywords:** delirium, intensive care units, pediatric, preschool, psychiatry, validation study

## Abstract

**Objective:** The goal of the present study was to perform a cross-cultural adaptation and clinical validation of the Preschool Confusion Assessment Method for the Intensive Care Unit–Spanish (psCAM-ICU-S) for its clinical use in the Colombian Population.

**Methods:** We designed a Cross-cultural adaptation study followed by a cross-sectional validation study at a Single-center Pediatric Intensive Care Unit (PICU) at a University Hospital in Bogotá, Colombia. The study population was children aged from 6 months to 5 years and 11 months who had been treated in the PICU with a Richmond sedation-agitation scale score of−3 or higher. A three-phase study was carried out. The first phase comprised the application of psychometric tests on the tool. In the second phase, the psCAM-ICU-S was applied to the target population. Patients were evaluated by a nurse and a pediatric intensivist using the psCAM-ICU-S; additionally, a child psychiatrist evaluated each patient using the *DSM-V* criteria; the psychiatrist evaluation was chosen as the gold standard for the diagnosis of delirium. In the third phase, an evaluation of the tool's effectiveness was carried out by using sensitivity, specificity, positive predictive value (PPV), negative predictive value (NPV), and likelihood ratios. Interrater agreement was also assessed by using the Fleiss' kappa.

**Results:** Psychometric tests established the instrument's reliability and consistency as well as the clarity of its items. A total of 31 patients were evaluated. On average, the instrument presented a sensitivity of 93.3%, specificity of 94.8%, PPV 78%, NPV 99%, a positive likelihood ratio of 19.93, and a negative ratio of 0.07. The prevalence of delirium was 16.1% by the child psychiatrist and 25.8% using de psCAM-ICU-S. We confirmed high Interrater agreement, Kappa index (0.672–0.902).

**Conclusions:** The psCAM-ICU-S was a valid and reliable instrument for the diagnosis of delirium in critically ill pediatric patients.

## Introduction

Delirium is an acute brain dysfunction characterized by an acute disturbed state of mind due to fluctuating mental status, inattention, and the inability to receive, process, or remember information (1). Mechanical ventilation, use of vasopressors, antiepileptics, and benzodiazepines as well as immobilization and age < 2 years are, among others, the main associated risk factors (2). In pediatrics, delirium has been better recognized in recent years. However, the condition remains underdiagnosed due to diagnostic limitations in pediatric patients (3). In addition to the team's lack of general knowledge about delirium, the lack of routine use of a diagnostic tool is a major barrier to diagnosis. A recent study carried out on three PICUs in Rio de Janeiro showed that the diagnosis of delirium in PICUs that did not use a valid diagnostic tool had a very low detection frequency (4). Diagnosis is relevant because pediatric delirium has shown negative consequences such as increased hospital stay, morbidity, and mortality as well as post-traumatic events in children (5–7). The prevalence in pediatric intensive care units (PICU) has been reported between 10 and 44% (2, 8–10).

There are various validated scales for the diagnosis of pediatric delirium in the English language, such as the Cornell Assessment of Pediatric Delirium (CAPD) (10), the Pediatric Confusion Assessment Method for the ICU (pCAM-ICU) in children 5 years of age and older (11), and the Preschool Confusion Assessment Method for the ICU (psCAM-ICU) for children under 5 years old, adapted from pCAM-ICU (8). The psCAM-ICU was translated into Spanish by Figueroa-Ramos et al. as Preschool Confusion Assessment Method for the Intensive Care Unit Spanish (psCAM-ICU-S) (12).

There is not a validated instrument in the Spanish language to assess delirium in patients under 5 years of age hospitalized in the PICU. There is an underestimation of the prevalence of delirium in pediatric patients due to the lack of validated instruments in this population (4). By validating this instrument, we hope to achieve a quick and accurate diagnosis, to guide medical treatment and perform interventions in patients with delirium. Additionally, it is necessary to have tools that allow identifying the predisposing factors and thus apply preventive measures. Therefore, the goal of the present study was to perform a cross-cultural adaptation for the Spanish spoken in Colombia given the cultural differences between the Spanish-speaking countries and to carry out a clinical validation of the psCAM-ICU-S for its clinical use in the PICU.

## Materials and Methods

This is a descriptive, observational study of cross-cultural adaptation and validation of the psCAM-ICU-S instrument, carried out at a University Hospital in Bogotá, Colombia, from April 20 to October 31, 2018. The study was developed in three phases. The first phase consisted of the development of psychometric tests supporting the use of the instrument. The second phase corresponded to the application of the instrument by the team of evaluators and the evaluation of the gold standard (child psychiatrist) for the diagnosis of delirium in critically ill preschool patients. Finally, in the third phase, the validity of the diagnostic test was determined by sensitivity, specificity, predictive values, and likelihood ratios.

### Phase 1: Psychometric Tests

To determine the face validity of the instrument, a group of seven experts participated in the study: a child psychiatrist, three pediatric intensivists, and four nursing specialists in pediatric critical care. The Lawshe index was used to establish the understandability of the items in the proposed instrument. Lawshe suggests that a content validity index (CVI) of at least 0.99 for each item would be necessary with the number of experts being seven or less (13). For this reason, any criteria with a lower value were modified. The survey applied to the group of professionals evaluated comprehension of each item as “understandable,” “not very understandable,” and “not understandable” according to the opinion and criteria of the experts. The items were grouped into “criteria” that were not evaluated as a set but did show an associated result according to the items that comprised each of them.

Seven experts evaluated the content validity according to sufficiency, clarity, coherence, and relevance. The concordance between evaluators was measured using the Fleiss Kappa. To evaluate its reliability, the instrument was applied to the eligible population in the PICU by the research team once the face and content validity had been established.

### Phase 2: Application of the Instrument to the Target Population

A translation of the psCAM-ICU instrument from English to Spanish was performed previously by Figueroa-Ramos et al. (12). The main investigator trained all the other participants, except for the child psychiatrist, in the application of the instrument. A meeting of the research team was held to clarify doubts in the data collection. Later, the instrument was applied to the target population by two nurses (surveyor 1 and 2), a pediatric intensivist (surveyor 3), and a child psychiatrist who set up a letter of commitment to guarantee the reliability of the data obtained in the application.

Spanish-speaking patients, aged between 6 months and 5 years and 11 months, hospitalized in the PICU and with a score higher than or equal to-3 on the Richmond Sedation and Agitation Scale (RASS), were included (14). Patients with visual and/or auditory alterations, delayed cognitive development, or in the terminal phase of the disease as well as those for whom the informed consent of the parents could not be obtained were excluded. A descriptive analysis was performed including the clinical and demographic characteristics of the patients.

The sample size calculation was performed by sample size for a given proportion. A sample size of 124 patients was obtained. However, when correcting and limiting the sample size by the total available population, a total sample size of 25 patients was obtained with an inflation of 15% for a final sample size of 29 patients.

Patients who met the inclusion criteria were evaluated by the research team. One of the nurses performed the first assessment of the patient with the instrument. Then the intensivist performed a second and independent assessment with the instrument. Finally, the child psychiatrist carried out a third evaluation based on the diagnostic criteria for neurocognitive disorders according to the *Diagnostic and Statistical Manual of Mental Disorders, Fifth Edition* (*DSM5*) (1) through a clinical assessment and a discussion with parents and/or caregivers to identify changes in the child's behavior. The data obtained in each of the evaluations were recorded in separate collection formats. The independent evaluations were performed no more than 3 h apart.

The prevalence of delirium was measured according to the gold standard by the child psychiatrist or, if the two evaluators agreed that the patient had delirium, according to the evaluation instrument. Concordance between evaluators was measured using the Fleiss Kappa, showing the level of agreement between the evaluators in a bivariate way, differentiating between each of the criteria and the general diagnosis. To demonstrate the reliability of the instrument, Cronbach's Alpha method was performed in each observation made by the experts as well as in the total of all the observations.

### Phase 3: Evaluation of the Instrument's Effectiveness

The final data from the application were collected by the psCAM-ICU-S instrument and the data from clinical assessment by the child psychiatrist. To determine the effectiveness of the psCAM-ICU-S, its ability to identify the presence or absence of delirium was established in comparison with evaluation in the Gold Standard reference method, with the measures of sensitivity, specificity, positive predictive value, negative predictive value, and plausibility reasons. Finally, the Chi-square independence tests were applied, which allowed to establish the level of association between the results provided by the instrument in each of its evaluators and the diagnosis provided by the psychiatrist. For statistical analysis, the Excel and R-Project were used to perform a data quality control, which made it possible to show whether there are atypical, anomalous data, or typing errors altering the quality and reliability of the results.

This study was approved by the ethics committee of the Fundación Santa Fe de Bogotá University Hospital. In all cases, informed consent was obtained from direct family members.

## Results

**Figure 1:**
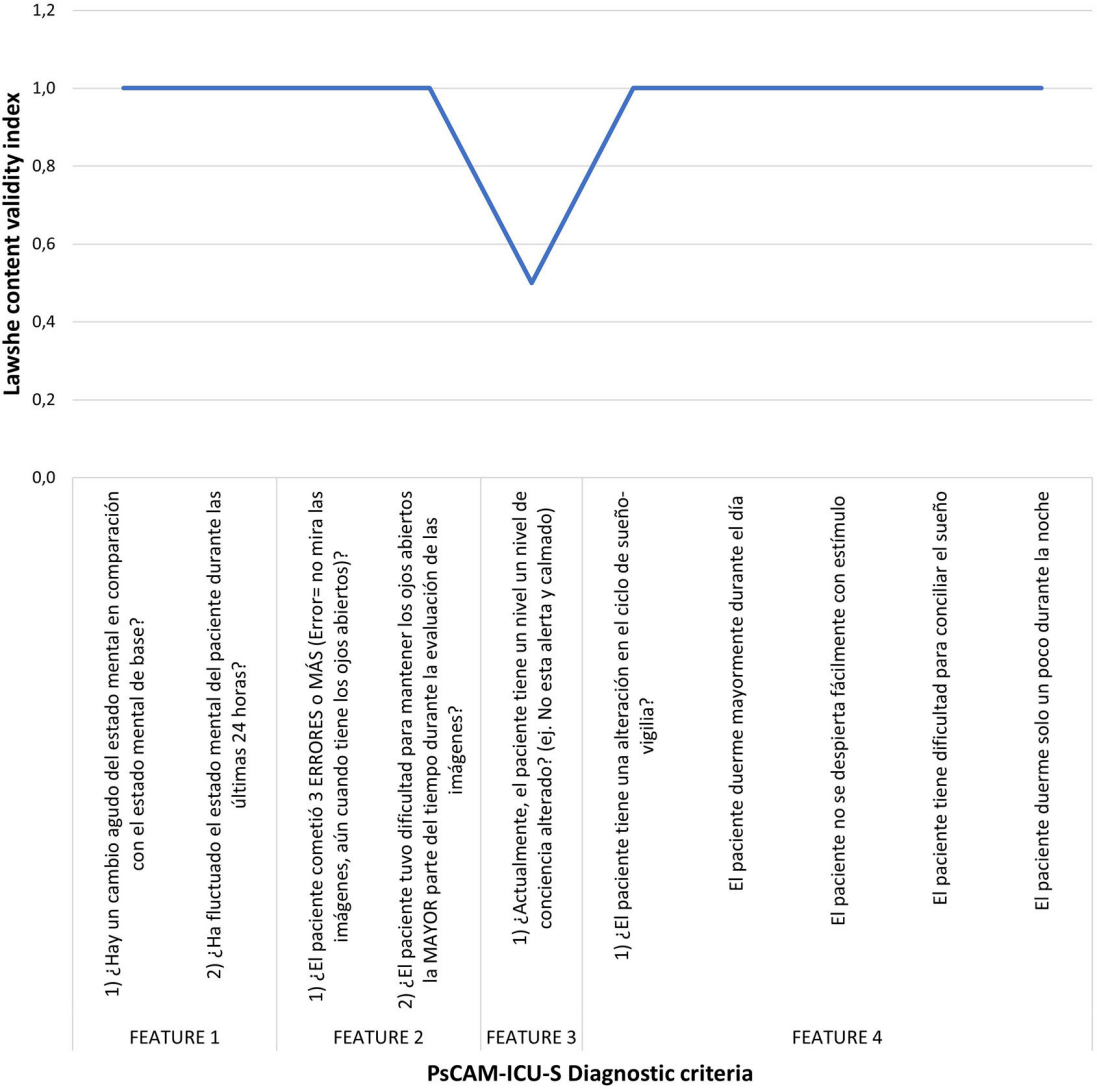
Lawshe index for psCAM-ICU-S criteria. All items obtained an adequate result except for feature 3 for which a modification was made.

Figure 1 summarizes the results obtained according to the Lawshe Index, calculated for the level of understanding of the items and the criteria considered in the instrument. Following the criterion proposed by Lawshe, it was possible to establish that all items and criteria are understandable (I. L = 1.0) except for criterion 3: Altered level of consciousness, made up of a single item that obtained a calculated Lawshe Index of 0.5. According to the observations of the expert who responded that it was “not very understandable” this item was modified as follows:

Original: ¿*Actualmente, el paciente tiene un nivel de conciencia alterado? (ej. no está alerta y calmado)*.
Modification: ¿*Tiene el paciente un nivel de conciencia alterado? (ej. no está alerta y calmado)*

For content validity, an average of 90% of the experts rated as “strongly in agreement” that the items were sufficient, clear, consistent, and relevant to the instrument. In addition, 9% rated “agree” with the aspects, and the remaining 1% on average rated “disagree” in any of the aspects. The results obtained from the kappa coefficient for each aspect evaluated are shown in Table 1. The final version of psCAM-ICU-S adapted to the Spanish spoken in Colombia is presented in Supplementary Material.

**Table 1:** The assessment of content validity.

**Aspect**	**Score**		**Kappa**	***P*-value**
**Sufficiency:** The surveyed items are enough	1	No: The item is not sufficient to meet the objective.	0.92	0.034
to meet the objective.	2	Low level: The items evaluate some of the aspects of the objective, but do not fully respond to it		
	3	Moderate level: If some items or options must be increased to meet the objective.		
	4	High level: If the items are enough.		
**Clarity:** The syntactic and semantics of	1	No: The item is not clear.	0.4	0.002
the item are adequate.	2	Low level: The item requires quite a few modifications or an exceptionally large modification in the use of words according to their meaning or the ordering of these.		
	3	Moderate level: Requires an extremely specific modification of some of the terms of the item.		
	4	High level: The item is clear, has adequate semantics and syntax.		
**Consistency:** The item is logically related to	1	No: The item has no logical relationship with the objective of the survey.	0.96	<0.001
the objective.	2	Low Level: The item has a tangential relationship with the target.		
	3	Moderate level: The item has a moderate relationship with the objective.		
	4	High level: The item is completely related to the dimension it is measuring.		
**Relevance:** The item is essential, that is, it must	1	No: The item can be deleted without affecting the measurement.	0.97	<0.001
be included.	2	Low level: The item has some relevance, but another item may be including what it measures.		
	3	Moderate level: The item is essential, that is, it must be included.		
	4	High level: The item is relevant and must be included		

The internal consistency between items using Cronbach's Alpha included 5 items for each evaluator, and the result of each criterion of the psCAM-ICU and the diagnosis of delirium by the instrument. This included 31 observations for each evaluator. The first expert presented a Cronbach's Alpha of 0.679, the second of 0.761, and the third of 0.696. Finally, for the calculation of global reliability, which included 5 items and 93 observations given by the 3 experts, a Cronbach's Alpha of 0.909 was obtained. Agreement between evaluators was measured through the Fleiss Kappa, in which the level of the agreement existing between the evaluators could be evidenced, making a distinction between each of the criteria and the general diagnosis. Table 2 shows the results obtained in the measurement of contrast between the evaluators.

**Table 2:** Fleiss's kappa of each criterion of psCAM-ICU-S and final diagnosis.

**Criterion**	**Evaluator 1 vs. 2**		**Evaluator 2 vs. 3**		**Evaluator 1 vs. 3**	
	**Kappa**	***P*-valor**	**Kappa**	***P*-valor**	**Kappa**	***P*-valor**
1	0.716	0.000	0.870	0.000	0.839	0.000
2	0.903	0.000	0.795	0.000	0.89	0.000
3	0.783	0.000	0.831	0.000	0.627	0.000
4	0.922	0.000	1	0.000	0.922	0.000
Final diagnosis	0.902	0.000	0.672	0.000	0.762	0.000

*Criterion 1: Cambio agudo o Curso fluctuante del estado mental. Criterion 2: Inatención. Criterion 3: Nivel de conciencia alterado. Criterion 4: Trastorno del ciclo Sueño-Vigilia. Final diagnosis: presence or absence of delirium*.

Fifty patients between the age of 6 months and 5 years were selected and admitted to the PICU; 31 of them met the inclusion criteria and signed the informed consent, and 19 were excluded. Finally, 31 patients were included, 12 females (38.7%) and 19 males (61.3%), aged between 6 months and 5 years and 11 months; the main admission diagnoses were a respiratory failure (42%), bronchiolitis (9.7%), asthma attack (6.45%), and acute respiratory infection (6.45%), and the remaining 35.4% were admitted with a primary diagnosis other than respiratory. The participants characteristics are shown in Table 3.

**Table 3:** Descriptive characteristics of parents and infants.

**Characteristics**	***n* = 31 (%)**
Gender	
Male	19 (38.7)
Female	12 (61.3)
Admission diagnosis	
Respiratory failure	13 (41.9)
Acute respiratory infection	7 (22.6)
Postoperative^*^	3 (9.7)
Acute asthma	2 (6.4)
Obstructive syndrome bronco	1 (3.2)
Recurrent wheezing syndrome	1 (3.2)
Abdominal septic shock origin	1 (3.2)
Polytrauma	1 (3.2)
Hemolytic uremic syndrome	1 (3.2)
Terminal ileum mechanical obstruction	1 (3.2)
Mechanical ventilation	5 (16.1)
	**Median (IQR)**
Age, months	24 (12–36)
PICU length of stay (d)	7 (5–9)

* *The three postoperative were of peritoneal shunt ventricle; release of abdominal adhesions; escharotomy and placement amniotic membrane*.

Each patient was evaluated on 4 occasions, 3 using the instrument and once by the child psychiatrist. In total, 93 evaluations were carried out using the instrument, and 31 evaluations by the child psychiatrist. According to the Gold standard, the prevalence of delirium in this population was 16.1% (*n* = 5), and according to the second criteria (a positive psCAM-ICU-S for delirium for two or more evaluators), 3 more patients (9.6%) were diagnosed with delirium, thus raising the prevalence to 25.8%. Of the eight patients who resulted positive for delirium, 62.5% presented hypoactive and 37.5% hyperactive delirium.

Three different evaluators classified patients by means of the instrument into patients with presence of and absence of delirium. Table 4 shows the sensitivity, specificity, negative predictive value, positive predictive value, likelihood ratios, and Chi-square of each of the evaluators. The sensitivity of the instrument in the 3 evaluators was between 80 and 100% with an average of 93.3% (95% CI, 95–96). Specificity values were between 92 and 96% with an average of 94.8% (95% CI 80–100). The positive predictive value was between 71 and 83%, with an average of 78% (95% CI 71–83). The negative predictive value was between 96 and 100%, with an average of 99% (95% CI 96–100). Finally, the positive likelihood ratio was between 13 and 26 with an average of 19.93 (95% CI 13–26) and the negative likelihood ratio between 0 and 0.21 with an average of 0.07 (0–0.21). This included Chi-square of 10.5 (*p* < 0.001).

**Table 4:** Validity of the pCAM-ICU-S instrument for the diagnosis of delirium.

**Statistic test**	**Evaluator 1**	**Evaluator 2**	**Evaluator 3**
Sensitivity %	80	100	100
Specificity %	96.15	92.31	96.15
Positive predictive value	80	71	83
Negative predictive value	96	100	100
Positive likelihood ratio	20.80	13.00	26.00
Negative likelihood ratio	0.21	0.00	0.00
Chi squared	13.84	11.39	6.27

## Discussion

Cross-cultural adaptation has two essential components: translation and adaptation (15). The process of cross-cultural adaptation implies developing versions of an assessment instrument that are equivalent to the original, but at the same time, linguistically and culturally adapted to a context different from the original. This method allows reducing costs and time in the preparation of new scales (16). Consequently, a linguistic process and adaptation of the instrument must be carried out using psychometric tests (15). We performed a cross-cultural adaptation of the psCAM-ICU-S for Spanish spoken in Colombia. Additionally, this study represents the first report of validation of the psCAM-ICU in the Spanish version. Although this instrument was translated into Spanish as psCAM-ICU-S, this version lacked formal validation (12). We found that the psCAM-ICU-S instrument showed a good concordance between observers, a good level of internal consistency, and an adequate validity when compared with the reference standard.

There are many bedside tools for delirium assessment. One of the most used is the Cornell Assessment of Pediatric Delirium (CAPD), which has been validated for use in children of all ages and has been implemented as a standard of care in many number of PICUs (17). In children with typical development, the CAPD is both sensitive and specific. However, in children with significant developmental delay, the CAPD has decreased specificity (10). CAPD requires anchor points for each stage of development, as it evaluates a range of behavior, from normal levels to varied levels of abnormal. Conducting this assessment appropriately takes time. In contrast, psCAM-ICU evaluates the lowest common denominator that would be considered abnormal for the broader cohort, thus allowing a faster evaluation. It is useful in ventilated and non-ventilated patients; it allows a rapid evaluation of delirium, taking 2 min to apply it (8). Since the psCAM-ICU was already translated into Spanish, unlike other scales such as the CAPD, which did not have the translation process, we decided to use the first one for adaptation and validation in our population.

Performing the Psychometric Tests of the psCAM-ICU instrument in the first phase it was established that criterion 3 was not understandable. After modification of this question, all the instrument's items were understandable. Regarding content validity, the kappa index of the 7 experts evidenced outstanding and almost perfect agreements (18). Finally, a reliability assessment by Cronbach's alpha with alpha values close to 0.7 in each of the expert observations and with a global value of 0.909 proved the instrument as reliable, with items well formulated and without mutual contradiction, and as consistent in measurement (19).

In the second phase, when applying the instrument to our population, the prevalence of delirium was 16.1% using the gold standard and 25.8% with the psCAMICU-S. According to the Chi-square tests, a strong association (*p* < 0.001) was established between the results provided by the instrument in each of its evaluators and the diagnosis provided by the psychiatrist. Compared to other studies using the same tool, our prevalence of delirium was lower. In the original study of the psCAM-ICU performed by Smith et al. with an assessment of 271 patients, a total delirium prevalence of 48% was found. However, there was a higher proportion of infants than in our study; when patients were subdivided into groups, and the prevalence of delirium decreased to 33% when they included only children under 2 years of age (8). Additionally, we only evaluated each patient in 1 day of stay in the PICU, and by not performing follow-ups we may have an undervaluation of the prevalence of delirium.

Finally, in the effectiveness evaluation, we obtained a specificity of 94.8%, a sensitivity of 93.3%, a PPV of 78%, and a NPV of 99%. These results were in line with those reported by the initial psCAM-ICU study (8) and validations carried out in other languages (20). Furthermore, the psCAM-ICU-S demonstrated excellent reliability with a kappa index of 0.78 (21). As to likelihood ratios the instrument's average classification would be “good;” this result is satisfactory due to the low proportion of patients with delirium in the critically ill pediatric population.

We acknowledge various limitations of the study. It was carried out in a single medical center, with a sample size being significant for our institution, but too small for any generalization of the results. Moreover, the onset of delirium can change with time, the time of the day, administered medications, and the patient's clinical baseline status. Therefore, variabilities could be seen between one assessment and the other. The time difference between the evaluations performed by the other evaluators and that made by the child psychiatrist was no more than 3 h to avoid changes in the state of consciousness. Still, the ps-CAM ICU requires the presence of inattention at the time of the scale to obtain the diagnosis of delirium. While the GOLD-standard as is the psychiatric evaluation assesses this presence of inattention both at the time of the interview and in patterns over a 24-h period, thus leading to the potential of additional delirium diagnoses (8). Also, each patient was assessed only four times, three using the scale and one by the child psychiatrist on a single day, so the prevalence of delirium may be underestimated since no follow-up was carried out in the remaining days.

The results of this study suggest that the Spanish version is easy to apply in Colombia and underwent a cultural adaptation process allowing it to be considered conceptually, semantically, and technically equivalent to the original version and with adequate psychometric characteristics after its application in a sample of patients between 6 months and 5 years 11 months of age. It is a tool that can be used in clinical practice as well as in research to evaluate the impact of interventions in preschool pediatric patients with delirium in the PICU. It is necessary to carry out prospective studies using the Spanish version for Colombia to determine the impact of the disease on our population.

## Data Availability Statement

The raw data supporting the conclusions of this article will be made available by the authors, without undue reservation.

## Ethics Statement

The studies involving human participants were reviewed and approved by Ethics committee Fundación Santa Fe de Bogotá University Hospital. Written informed consent to participate in this study was provided by the participant's legal guardian/next of kin.

## Author Contributions

EP-C, MS-T, AH-C, and NG contributed to the design of the study, data collection, and data analysis. LC-A contributed to data collection, data analysis, and drafted the first manuscript. All authors read and approved the final manuscript.

## Conflict of Interest

The authors declare that the research was conducted in the absence of any commercial or financial relationships that could be construed as a potential conflict of interest.

## Publisher's Note

All claims expressed in this article are solely those of the authors and do not necessarily represent those of their affiliated organizations, or those of the publisher, the editors and the reviewers. Any product that may be evaluated in this article, or claim that may be made by its manufacturer, is not guaranteed or endorsed by the publisher.
