# Supplementary material for: Preschool Confusion Assessment Method for the Intensive Care Unit–Spanish (psCAM-ICU-S): Cross-Cultural Adaptation and Validation in Colombia
Source: Front Pediatr. 2021 Dec 7;9:749522. doi: 10.3389/fped.2021.749522 (PMC8688817; doi:10.3389/fped.2021.749522)
Supplement: Supplementary file 1 [file Image_1.pdf]

Supplementary material

Supplementary material 1. Final version of psCAM-ICU-S adapted to the Spanish spoken in Colombia

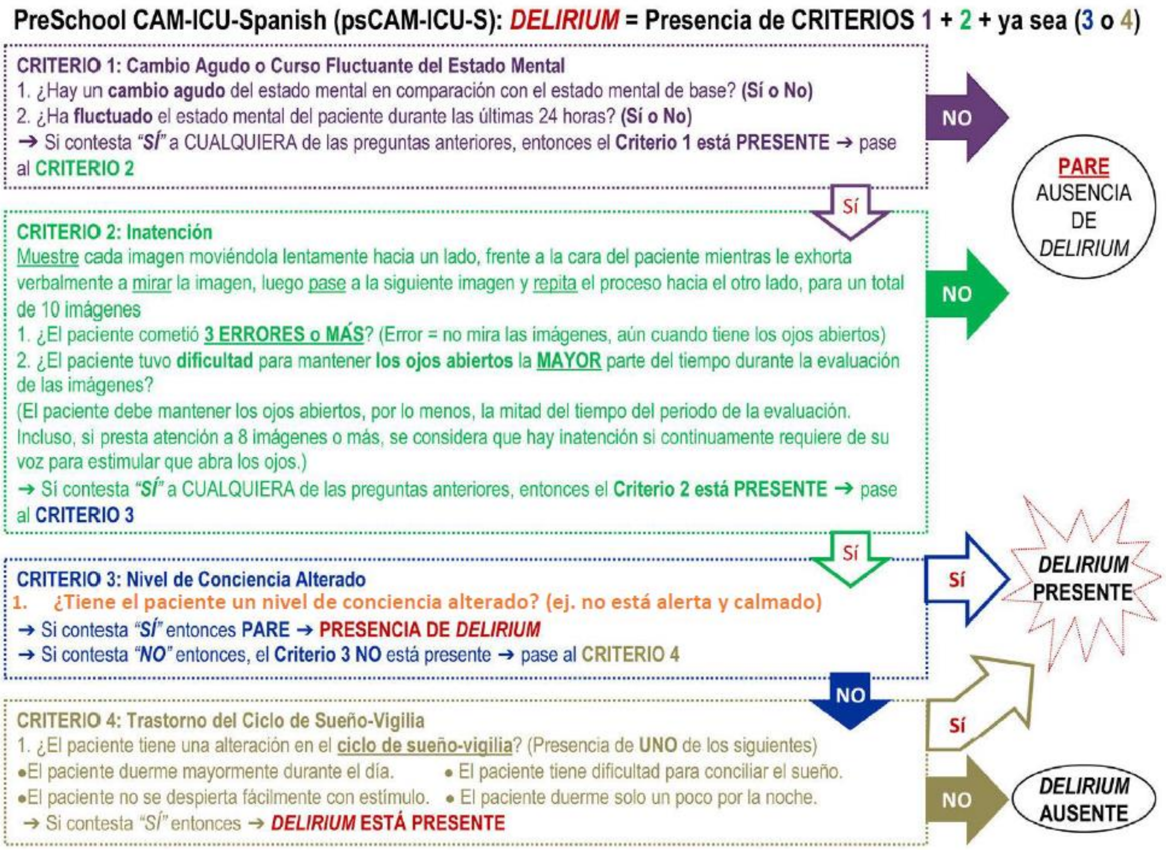

The only change made to the original tool translated into Spanish was in criterion 3, change highlighted in orange. Adapted from: Figueroa-Ramos MI, Arroyo-Novoa CM, García-De Jesús RL, Sepúlveda-Santiago CS, Solís-Báez SS, Ely EW, et al. Traducción y adaptación cultural al español del Preschool Confusion Assessment Method for the Intensive Care Unit. Med Intensiva. 2020 Oct 1;44(7):453–6
